# Mechanisms by Which Exercise Alleviates Depressive Symptoms: The Potential Role of Short-Chain Fatty Acids

**DOI:** 10.3390/nu18203311

**Published:** 2026-10-09

**Authors:** Qin Zhang, Jiheng Yuan, Zhengrui Wang, Jing Miao, Hongyu Gong, Yumei Han

**Affiliations:** School of Physical Education, Shanxi University, Taiyuan 030006, China; sxdx1221@163.com (Q.Z.); yuanjiheng@sxu.edu.cn (J.Y.); 13835658310@163.com (J.M.); hongyu025@163.com (H.G.)

**Keywords:** depression, exercise, gut microbiota, short-chain fatty acids, gut microbiota–brain axis

## Abstract

Short-chain fatty acids (SCFAs) are important metabolic products generated by the gut microbiota and they play a crucial role in maintaining intestinal barrier integrity, regulating immune responses, and participating in neural modulation. In recent years, accumulating evidence has indicated that dysregulated SCFA levels are closely associated with depressive states and the onset and progression of depression. SCFAs may be involved in the pathophysiology of depression through multiple pathways, including modulation of gut microbiota homeostasis, inflammatory responses, vagal nerve signaling, and 5-hydroxytryptamine (serotonin) metabolism. This review summarizes the roles of SCFAs in depressive states and depression, the regulatory effects of different exercise modalities on SCFA metabolism, and the potential mechanisms by which exercise-mediated SCFA modulation alleviates depressive symptoms, aiming to provide a theoretical basis for exercise-based interventions in depression.

## 1. Introduction

Depression is a common affective psychiatric disorder and represents a substantial global public health burden. According to the latest estimates reported by the World Health Organization (WHO), approximately 322 million people worldwide were living with depression in 2023, accounting for approximately 4% of the global population and 5.2% of adults [1]. The gut microbiota of individuals with depression is particularly susceptible to dysbiosis in response to internal and external environmental factors. Such dysbiosis not only increases susceptibility to the disorder but also disrupts metabolic and immune functions and exerts adverse effects on the brain and central nervous system [2].

The clinical relevance of exercise in depression is also reflected in current guidelines and professional recommendations. The National Institute for Health and Care Excellence (NICE) includes structured group exercise among the treatment options for adults with depression [3]. The World Health Organization (WHO) recommends encouraging physical activity as part of the treatment of adults with depressive disorders, particularly those with inactive lifestyles, and considers physical activity as an adjunct to antidepressant medication or structured psychological interventions in moderate-to-severe depression [4]. In addition, guidance from the European Psychiatric Association (EPA) supports physical activity as an evidence-based component in the management of major depressive disorder [5].

**Figure 1:**
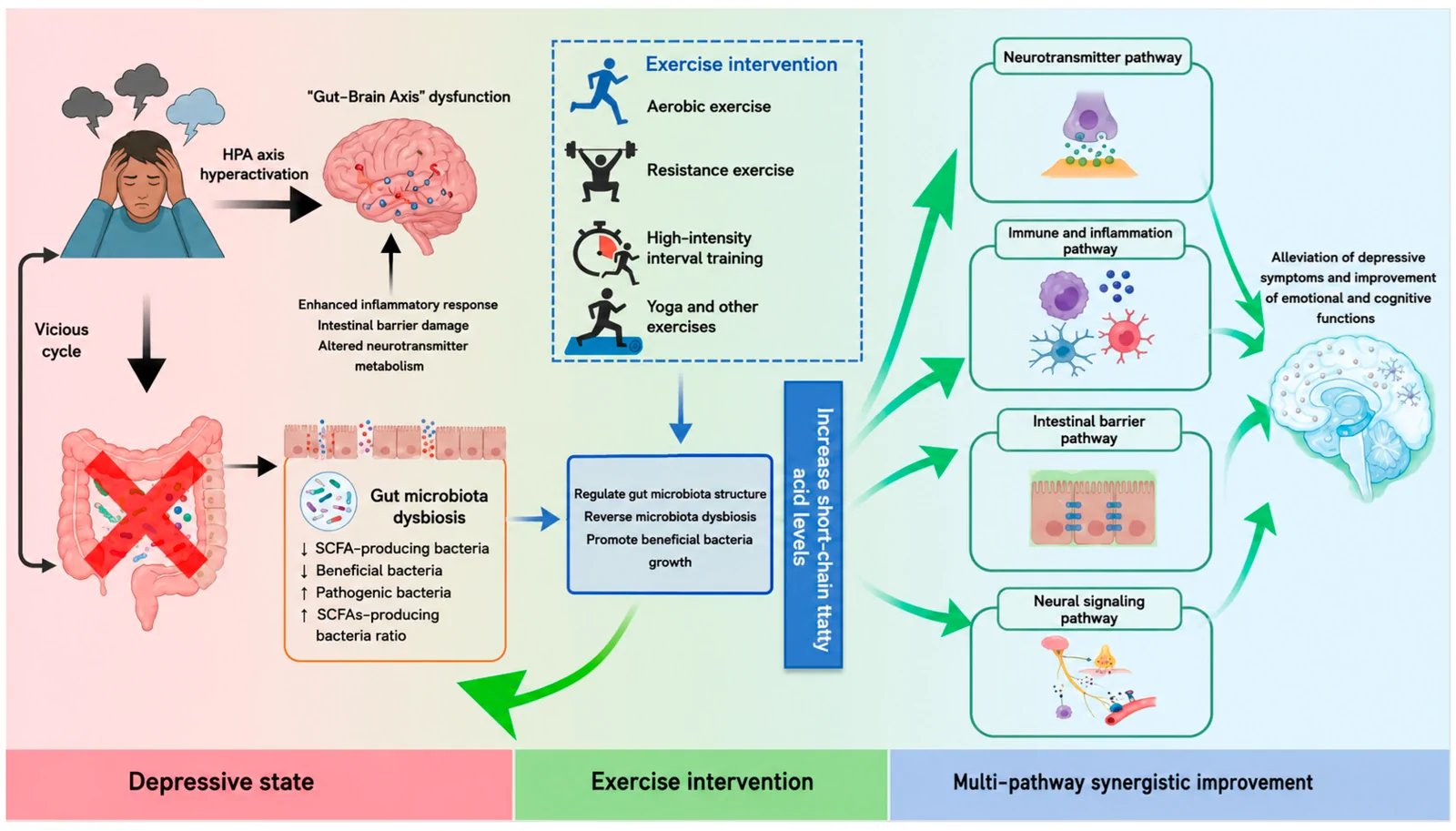
Mechanisms by which exercise alleviates depressive symptoms: the potential role of short-chain fatty acids.

Short-chain fatty acids (SCFAs) are major metabolites produced by the gut microbiota through the fermentation of dietary fiber and other carbohydrates, primarily including acetate, propionate, and butyrate. SCFAs contribute to the maintenance of intestinal barrier integrity, regulate inflammatory and immune responses, and influence central nervous system function through the systemic circulation and neural pathways. Accumulating evidence has demonstrated that abnormal SCFA levels are closely associated with depressive states and the onset and progression of depression. SCFAs may participate in the pathophysiology of depression by modulating inflammatory responses, vagal signaling, neurotransmitter synthesis, and the function of the gut microbiota–brain axis. As an important non-pharmacological intervention, exercise may alleviate depressive symptoms by improving the composition of the gut microbial ecosystem, increasing the abundance of SCFA-associated bacteria, and modulating SCFA-related microbial metabolism. This review summarizes current advances in research on the role of exercise-mediated regulation of SCFAs in alleviating depressive symptoms, thereby providing a theoretical basis for the development and clinical application of exercise prescriptions for depression. The potential role of SCFAs in the antidepressant effects of exercise is summarized in Figure 1.

## 2. Overview of Short-Chain Fatty Acids

Short-chain fatty acids are a class of fatty acids containing no more than six carbon atoms that are produced by the gut microbiota through the fermentation of dietary components in the intestine. As important metabolites within the intestinal ecosystem, SCFAs not only serve as a major energy source for intestinal epithelial cells but also protect intestinal health by regulating inflammatory responses and maintaining intestinal barrier integrity [6].

Within the intestinal microenvironment, the concentrations and relative proportions of different SCFAs are not constant but are jointly influenced by multiple factors, including gut microbial composition, dietary nutrient composition, and intestinal transit time [7]. SCFAs produced by gut microorganisms can lower the luminal pH, thereby creating a favorable environment for the growth and proliferation of beneficial bacteria and promoting the maintenance of gut microbial homeostasis, ultimately contributing to intestinal health [8].

Most SCFAs are utilized locally within the intestine, whereas a smaller proportion enters the systemic circulation through monocarboxylate transporters (MCTs) and sodium-coupled monocarboxylate transporters (SMCTs) [6,9]. SCFAs also act as endogenous ligands for free fatty acid receptors (FFARs), which are widely expressed on the surface of cells in the intestinal endocrine, immune, and nervous systems. By binding to these receptors and initiating downstream biological signaling, SCFAs can influence behavior and emotional states through neuroendocrine and immune signaling pathways [10].

Alterations in SCFA metabolism have been increasingly associated with depression. A clinical study reported differences in fecal SCFA profiles between women with depression and healthy women, suggesting that depression may be associated with alterations in gut microbial SCFA metabolism [11]. However, the direction and magnitude of these alterations may vary among individual SCFAs and across different biological compartments.

SCFAs may interact with the central nervous system through the systemic circulation and monocarboxylate transport systems [12]. SCFAs can also contribute to intestinal barrier integrity and exert anti-inflammatory effects through multiple signaling mechanisms [6]. Sodium butyrate administration has been reported to alleviate depressive- and manic-like behaviors in animal models [13]. Fecal microbiota transplantation has been reported to improve gastrointestinal and psychological symptoms in patients with diarrhea-predominant irritable bowel syndrome accompanied by anxiety and depression, together with changes in gut microbial metabolism and fecal SCFA levels [14]. Although these findings do not establish a direct causal role for SCFAs in depression, they further support a potential relationship between gut microbial metabolism, SCFAs, and depressive symptoms. Importantly, SCFA dysregulation may involve not only changes in total concentrations but also alterations in the relative composition of individual SCFAs. Given the distinct metabolic and physiological functions of acetate, propionate, and butyrate, both their absolute concentrations and relative proportions should be considered when evaluating SCFA dysregulation in depression.

### 2.1. SCFAs Regulate Gut Microbial Dysbiosis in Depression

SCFAs comprise a diverse group of microbial metabolites generated through the fermentation of dietary substrates. Dietary fiber that escapes digestion in the upper gastrointestinal tract can be utilized by gut microorganisms and fermented through multiple metabolic pathways, leading to the production of SCFAs, primarily acetate, propionate, and butyrate. These SCFAs are subsequently absorbed and utilized by the host and participate in multiple physiological processes. In particular, SCFAs serve as important energy substrates within the intestinal ecosystem and contribute to the maintenance of colonic health and intestinal barrier integrity [15].

Although acetate, propionate, and butyrate are often discussed collectively as SCFAs, they differ substantially in their metabolic fate, signaling properties, and biological functions. Acetate is the most abundant SCFA in the circulation and serves as an important substrate for peripheral metabolism while also participating in GPCR-mediated metabolic and immune signaling [16]. Propionate is largely metabolized by the liver and is involved in gluconeogenic metabolism, immune regulation, and mucosal homeostasis [16,17,18]. Butyrate is preferentially utilized as an energy substrate by colonocytes and plays a particularly important role in maintaining intestinal epithelial integrity; it also exerts anti-inflammatory and epigenetic effects through GPCR activation and HDAC-related mechanisms [19]. Importantly, propionate and butyrate do not necessarily exert identical biological effects, and their downstream metabolic, epigenetic, and physiological actions may differ substantially [17,19]. These functional differences indicate that individual SCFAs should be considered separately when interpreting SCFA dysregulation in depression and exercise-induced changes in SCFA metabolism.

The gut microbiota differs markedly between healthy individuals and patients with depression. Clinical studies have identified alterations in gut microbial composition in patients with depression, although the findings for microbial richness and alpha diversity have not been entirely consistent across different populations. Some studies have reported reduced microbial richness or diversity, whereas others have primarily observed changes in the relative abundance of specific bacterial taxa. These inconsistencies may be related to differences in dietary patterns, medication use, disease severity, geographic background, sequencing methods, and sample size. Nevertheless, the available evidence generally supports the presence of gut microbial dysbiosis in at least a subset of patients with depression [20]. Several studies have reported alterations in bacterial taxa associated with SCFA production in patients with depression. Reductions in certain butyrate-producing bacteria, including members of Faecalibacterium, Roseburia, and related taxa within the family Lachnospiraceae, have been observed in some cohorts. However, these microbial alterations are not uniform across all studies, and changes in the abundance of SCFA-associated bacteria do not necessarily correspond directly to fecal or circulating SCFA concentrations [21]. Changes in dietary composition can alter the diversity and abundance of the gut microbiota, which may further regulate SCFA production and metabolism and consequently contribute to the alleviation of depressive symptoms [22].

Animal studies have shown that prolonged consumption of a high-fat, low-fiber diet impairs the capacity of the gut microbiota to produce SCFAs and may induce systemic inflammation. These inflammatory mediators can compromise the integrity of neuronal cell membranes and suppress the expression of neurotrophic factors in key brain regions, including the hippocampus and cerebral cortex [23]. In studies involving exogenous SCFA supplementation in depressive rat models, sodium butyrate significantly alleviated depressive-like behaviors in some experiments. By contrast, although sodium acetate, sodium propionate, and SCFA mixtures altered gut microbial composition, their antidepressant effects were not significant [24].

SCFAs can also reduce stress-induced corticosterone release, suggesting that they play an important role in regulating stress responses and alleviating stress. In addition to improving the intestinal microenvironment, SCFAs can markedly reverse impairments in sociability and improve social behavior [25]. Gut microbial dysbiosis in patients with depression may alter SCFA production and the relative composition of individual SCFAs, whereas SCFA deficiency may further aggravate microbial imbalance, thereby creating a vicious cycle. Different SCFAs may influence depressive symptoms by regulating gut microbial composition, intestinal barrier function, and neuroimmune and neuroendocrine signaling. These findings provide a theoretical basis for the prevention and treatment of depression through targeted modulation of the gut microbiota–SCFA axis.

Recent systematic reviews and meta-analyses further indicate that depressive disorders are associated with alterations in gut microbial composition, although considerable heterogeneity exists across studies. Several SCFA-associated taxa, particularly butyrate-producing bacteria such as Faecalibacterium, Butyricicoccus, and Coprococcus, have been reported to be depleted in individuals with depression, whereas taxa such as Eggerthella, Enterococcus, and Streptococcus tend to be enriched. These alterations suggest that depression-associated dysbiosis may involve a shift in microbial communities supporting SCFA-related fermentation, particularly butyrate production. However, changes in bacterial abundance should not be interpreted as direct evidence of altered SCFA production, because microbial metabolic output is also influenced by substrate availability, cross-feeding interactions, strain-level functional differences, and host factors. The major microbial alterations reported in recent systematic reviews and meta-analyses and their potential relevance to SCFA metabolism are summarized in Table 1.

### 2.2. SCFAs Regulate Vagal Dysfunction in Depression

The vagus nerve is one of the principal pathways through which the gut microbiota communicates with the central nervous system. SCFAs can activate FFAR3 expressed on vagal neurons, thereby transmitting signals to the nucleus tractus solitarius in the brainstem and subsequently to the amygdala and prefrontal cortex. These brain regions play essential roles in the processing and regulation of emotion [10]. SCFA-mediated vagal signaling may further influence brain function by modulating peripheral immune responses. This mechanism illustrates how gut microbial metabolites can affect the central nervous system through integrated neuroimmune pathways [31].

SCFAs act on the intestine to stimulate L cells and enterochromaffin cells to release glucagon-like peptide-1(GLP-1) and 5-hydroxytryptamine (5-HT), respectively. These mediators activate vagal afferents and induce neuronal excitation, thereby transmitting intestinal signals to the central nervous system. This signaling pathway may increase brain 5-HT levels, reduce the abundance of pro-inflammatory cytokines such as tumor necrosis factor-α (TNF-α) and interleukin-6 (IL-6), and maintain cerebral energy homeostasis, ultimately exerting antidepressant effects [32].

SCFAs can also act as physiological regulators of the enteric nervous system by directly interacting with nerve endings, thereby enabling intestinal signals to rapidly evoke vagal responses. This direct mechanism suggests that SCFAs can modulate neural signaling rapidly and independently. In particular, butyrate can directly and rapidly stimulate the vagus nerve to generate neural electrical activity [33]. SCFAs can also stimulate intestinal L cells to secrete glucagon-like peptides and other mediators, which indirectly influence the central nervous system through vagal signaling. In response to these signals, the central nervous system regulates neurotransmitter and energy metabolism while suppressing inflammatory responses, thereby contributing to the alleviation of depressive symptoms [34].

A study found that transplantation of fecal microbiota from mice susceptible to chronic social defeat stress into antibiotic-treated wild-type and Ephx2-knockout mice induced an anhedonia-like phenotype in the recipient animals. These findings indicate that alterations in the gut microbiota can influence behavioral and neurophysiological states through the gut microbiota–brain axis [20]. Collectively, the available evidence suggests that the effects of gut microbial disturbances on depression may be mediated, at least in part, by the vagus nerve. The ability of SCFAs to modulate depressive symptoms through this pathway further supports the pivotal role of vagal signaling in gut microbiota–brain axis communication.

### 2.3. SCFAs Regulate Aberrant Inflammatory Responses in Depression

In key brain regions of patients with depression, including the hippocampus, prefrontal cortex, and amygdala, excessive microglial activation and astrocytic dysfunction are frequently observed. These pathological alterations can increase the release of pro-inflammatory cytokines, such as tumor necrosis factor-α (TNF-α) and interleukin-6 (IL-6). These inflammatory mediators not only impair neuroplasticity but also disrupt the normal physiological functions of neural cells, ultimately contributing to typical depressive symptoms, including low mood and cognitive impairment [35].

The gut microbiota can regulate host immune responses and neurotransmitter levels through the production of microbial metabolites and the release of inflammatory mediators. These effects can subsequently alter nervous system function and thereby contribute to the onset and progression of depression [36]. Studies have shown that neuroinflammation mediated by pro-inflammatory cytokines, including interleukin-1β (IL-1β), IL-6, and TNF-α, induces detrimental responses within the central nervous system and exacerbates depressive symptoms [37].

The integrity of the intestinal mucosal barrier is influenced by SCFA production. SCFAs not only help maintain gut microbial homeostasis but also modulate immune function and reduce the release of pro-inflammatory mediators, thereby playing an important role in alleviating depressive symptoms. Animal studies have shown that a high-fructose diet can disrupt the gut microbiota and intestinal barrier, induce hippocampal neuroinflammation, and increase the number of activated microglia in the hippocampus. SCFA supplementation exerts protective effects by restoring intestinal barrier integrity and attenuating neuroinflammation through modulation of inflammasome activity, thereby protecting neurons [38].

SCFAs can reduce inflammatory mediator production and help maintain intestinal homeostasis by inhibiting histone deacetylases (HDACs) and promoting the expression of genes and transcription factors involved in tight junction protein regulation [39]. SCFAs can also directly modulate immune cells, thereby controlling the production and release of inflammatory mediators and maintaining immune homeostasis. This mechanism is important for preventing excessive immune activation and the development of inflammatory responses [40].

In addition to directly regulating the secretion of inflammatory mediators by immune cells to maintain immune balance, oral administration of propionate and butyrate can increase the number of peripheral regulatory T cells in mice. As a key component of the immune system, regulatory T cells can effectively suppress excessive immune responses and thereby preserve immune homeostasis [41]. SCFAs may regulate aberrant inflammatory responses in depression by restoring intestinal barrier integrity and preventing the initiation of gut-derived inflammation, modulating peripheral immune cells and central microglia to limit the propagation of inflammation, and regulating inflammation-related signaling pathways to restore neuroimmune and neuroendocrine homeostasis [42].

### 2.4. SCFAs Regulate Serotonergic Dysregulation in Depression

Neurotransmitter imbalance is one of the hallmark features of depression, particularly the reduced availability of monoamine neurotransmitters such as 5-hydroxytryptamine (5-HT) and norepinephrine (NE), which is widely considered an important contributor to the development of depression. These monoamine neurotransmitters play indispensable roles in the regulation of mood, cognition, and behavior. Most antidepressant medications exert their therapeutic effects by increasing monoamine neurotransmitter levels, thereby alleviating depressive symptoms [43].

Central 5-HT is considered a representative biomarker associated with depressive states. Reduced 5-HT availability within the synaptic cleft is widely regarded as an important factor contributing to the development of depression. As a major neurotransmitter, 5-HT plays a central role in mood regulation, cognitive function, and behavioral control; therefore, decreased 5-HT levels may contribute to typical depressive symptoms, including low mood and loss of interest [44]. Gut microbiota-derived metabolites may also influence serotonergic signaling. In mice exposed to chronic unpredictable mild stress, administration of Clostridium butyricum alleviated depressive-like behaviors and modulated gut–brain axis-related alterations [45]. These findings suggest that modulation of the gut microbiota may influence neurobiological processes associated with depressive-like behavior.

**Figure 2:**
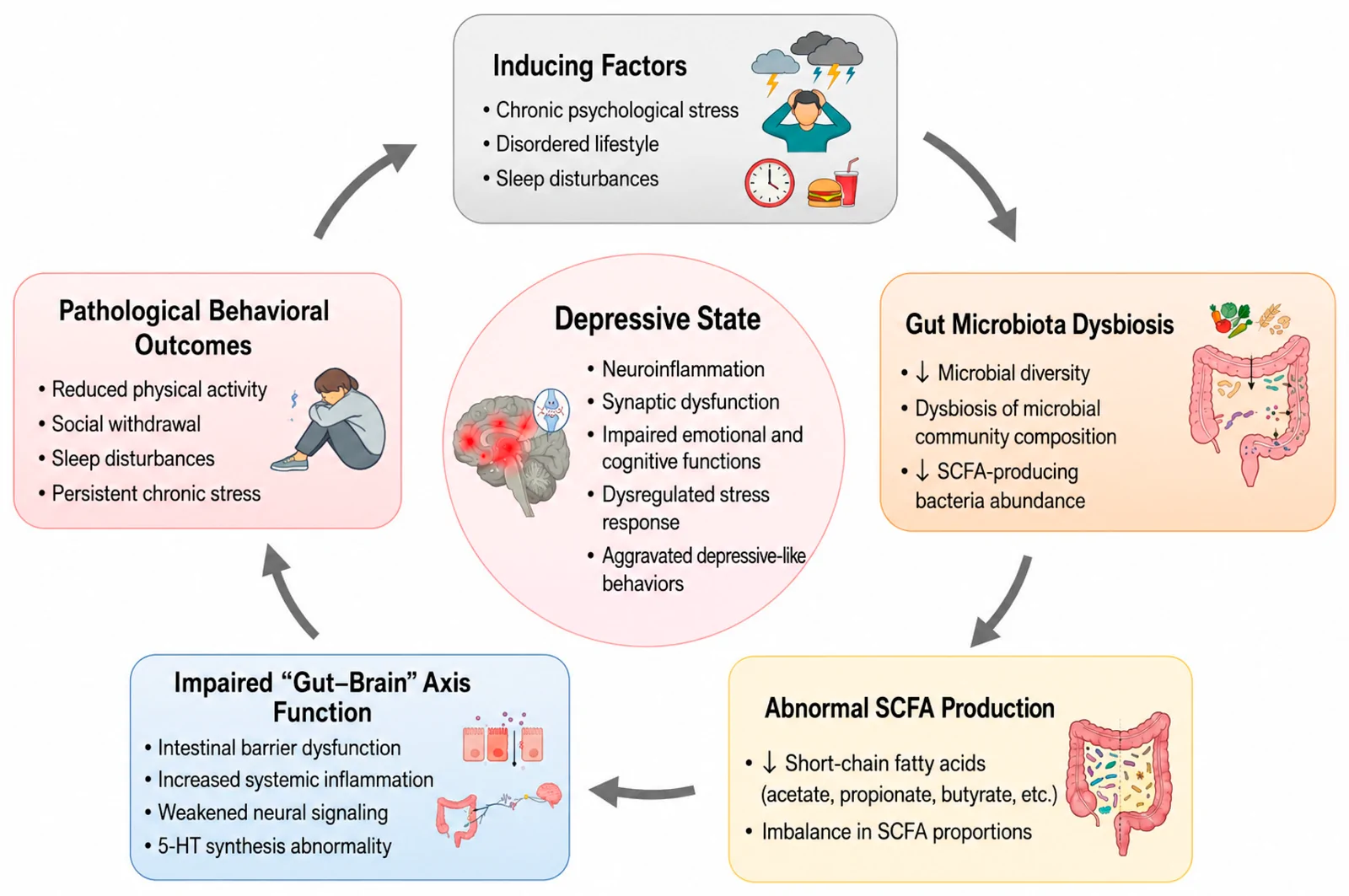
Short-chain fatty acid dysregulation in depression and its effects on the gut microbiota–brain axis.

SCFAs may provide a metabolic link between the gut microbiota and serotonergic signaling. Reigstad et al. [21] demonstrated that microbial SCFAs promote colonic serotonin (5-HT) production by stimulating enterochromaffin cells and increasing the expression of tryptophan hydroxylase 1 (TPH1), the rate-limiting enzyme in peripheral 5-HT synthesis. These findings support a role for SCFAs in the regulation of peripheral serotonergic metabolism. In addition, SCFAs may influence neuroplasticity and serotonergic signaling. In mice exposed to chronic unpredictable mild stress, sodium butyrate exerted antidepressant-like effects accompanied by alterations in brain-derived neurotrophic factor (BDNF)- and 5-hydroxytryptamine (5-HT)-related signaling [46]. These findings suggest that butyrate may influence depressive-like behavior, at least partly, through mechanisms involving neuroplasticity and serotonergic regulation. The major pathways through which SCFA dysregulation may contribute to depression are summarized in Figure 2.

## 3. Physical Activity as a Regulator of SCFA Production

Accumulating evidence indicates that physical activity can modulate the metabolic output of the gut microbiota, with SCFAs representing one of the microbial metabolite classes that are particularly responsive to exercise. However, current findings do not support a simple assumption that exercise uniformly increases SCFA production. Considerable heterogeneity has been observed in the responses of acetate, propionate, and butyrate across studies, suggesting that exercise-induced changes in SCFAs may depend on exercise intensity, duration, training modality, timing, host metabolic status, and baseline exercise capacity. Therefore, rather than considering exercise as a general enhancer of SCFA production, it may be more appropriate to view physical activity as a context-dependent regulator of microbial fermentation and SCFA metabolism.

### 3.1. Evidence Linking Physical Activity to SCFA Production

Accumulating evidence indicates that physical activity can alter intestinal SCFA production; however, this response is not uniform and appears to depend on exercise dose, intensity, modality, timing, host metabolic status, and baseline exercise capacity. Aerobic exercise currently provides the largest body of evidence supporting an exercise–gut microbiota–SCFA relationship. Beyond its established cardiovascular and psychological benefits [47], repeated aerobic exercise can modify both the composition and metabolic activity of the intestinal microbiota. Lambert et al. [48] subjected 6-week-old db/db mice to low-intensity treadmill exercise for 6 weeks, with three 60-min sessions per week, and observed increased fecal abundance of lactic acid bacteria and Faecalibacterium prausnitzii. These taxa are closely associated with microbial fermentation and SCFA metabolism, suggesting that even relatively low-intensity aerobic exercise may shift the intestinal ecosystem toward a greater SCFA-producing potential.

Exercise-induced microbial remodeling may involve taxa with different SCFA-related metabolic functions. For example, Faecalibacterium prausnitzii is a prominent butyrate producer, whereas Bifidobacterium primarily produces acetate and lactate during carbohydrate fermentation. Akkermansia muciniphila is mainly associated with acetate and propionate production through mucin utilization, while several members of Blautia and Roseburia contribute to acetate- and/or butyrate-related fermentation. These distinctions are important because an exercise-induced increase in a particular bacterial taxon does not necessarily predict a uniform increase in total SCFAs but may instead favor specific SCFA profiles.

Evidence from populations with metabolic disorders provides further support for this relationship. Torquati et al. [49] reported that aerobic exercise altered the intestinal abundance of Bifidobacterium, Escherichia, and butyrate-associated bacterial populations in patients with type 2 diabetes mellitus (T2DM). Consistent with these microbial changes, aerobic exercise has also been associated with increased acetate, propionate, and butyrate concentrations, indicating that exercise may influence not only microbial composition but also microbial metabolic output [50]. Similarly, Abraham D et al. [51] found that 20 weeks of treadmill exercise increased the abundance of butyrate-producing bacteria in mice, accompanied by changes associated with reduced inflammation and amelioration of Alzheimer’s disease-related abnormalities. These findings are relevant because they suggest that SCFA-related adaptations to exercise may extend beyond the intestine and contribute to systemic and neurological outcomes.

Other animal studies further demonstrate that exercise-induced SCFA responses can occur alongside substantial restructuring of the gut microbial community. Li et al. [52] reported that 4 weeks of daily treadmill training increased the intestinal abundance of Turicibacter and Romboutsia, reduced Helicobacteraceae, and increased SCFA production in rats. These alterations were accompanied by attenuation of intestinal inflammation and improvements in systemic immune and cardiovascular-related functions. Collectively, these studies indicate that aerobic exercise may promote SCFA production through coordinated changes in bacterial taxa and microbial metabolic activity rather than through expansion of a single SCFA-producing population.

Importantly, human intervention studies indicate that these adaptations are dynamic and may depend on continued exercise exposure. Following 6 weeks of aerobic endurance training, exercise-induced increases in fecal propionate and butyrate, butyrate-producing bacteria, and the relative abundance of the butyryl-CoA:acetate CoA-transferase (BCoAT) gene returned toward baseline after participants resumed a sedentary lifestyle for another 6 weeks [53]. This reversibility suggests that the SCFA-related microbial phenotype induced by exercise may require sustained physical activity and should not necessarily be regarded as a permanent restructuring of the gut ecosystem.

Studies of highly trained athletes also provide insight into microbial metabolic adaptations associated with repeated exercise. In professional marathon runners and Olympic rowers, exercise was associated with enrichment of Veillonella and an increased microbial functional capacity for lactate-to-propionate conversion [54]. This observation is particularly noteworthy because it shifts the focus from taxonomic composition alone to microbial substrate utilization and metabolic cross-feeding. Exercise may therefore modify SCFA production not only by increasing conventional SCFA-producing bacteria, but also by altering the availability and microbial utilization of exercise-associated metabolites.

Not all available evidence, however, provides direct confirmation of increased SCFA production. For example, a randomized controlled trial protocol proposed a 6-month Baduanjin intervention in individuals with prediabetes to examine glycemic control and gut microbial composition, but the protocol did not report completed intervention outcomes and therefore cannot be considered direct evidence that Baduanjin increases SCFA-producing bacteria or SCFA concentrations [55]. This distinction is important because studies reporting microbial compositional changes without directly measuring SCFAs cannot establish enhanced SCFA production. Nevertheless, because Bacteroidetes and other intestinal bacterial groups contribute substantially to acetate and propionate generation, exercise-induced shifts in these microbial populations may influence the overall fermentative capacity of the gut. Wei et al. [56] further demonstrated that aerobic exercise stimulated SCFA-related microbial metabolism, supporting the view that exercise can regulate microbial fermentation as well as microbial composition.

Taken together, the available evidence supports an association between aerobic exercise and enhanced SCFA-related microbial metabolism, but the magnitude and profile of this response are not uniform. Changes in bacterial abundance should therefore not automatically be interpreted as changes in SCFA production. Direct SCFA measurements, microbial functional genes, and metabolic pathway analyses are needed to distinguish taxonomic remodeling from genuine alterations in microbial fermentative output.

### 3.2. Exercise Modality and Intensity Determine SCFA Responses

Although aerobic exercise has been investigated most extensively, SCFA responses may differ substantially according to exercise modality. Resistance exercise provides a distinct physiological stimulus characterized by repeated skeletal muscle contractions against external loads and pronounced alterations in substrate utilization, energy expenditure, and systemic metabolism [57]. These host responses may indirectly modify the intestinal environment and microbial metabolic activity. Several studies have therefore proposed that resistance exercise can influence gut microbial composition and SCFA-related metabolism, although the evidence remains considerably more limited than that available for aerobic exercise [58].

In an animal study, Ma et al. [59] subjected 8-week-old mice to 8 weeks of progressively loaded ladder-climbing exercise. Resistance training increased muscular strength and significantly elevated serum acetate and butyrate concentrations, suggesting that resistance exercise may influence gut-derived metabolites. Nevertheless, circulating SCFA concentrations reflect the combined effects of intestinal production, absorption, tissue utilization, and clearance. Thus, increased serum SCFAs should not be interpreted as direct evidence of increased intestinal SCFA production without corresponding fecal or microbial functional measurements.

This limitation is particularly relevant when interpreting the human literature. A rapid review by Wagner et al. [60] concluded that studies investigating resistance exercise and the gut microbiota remain few and methodologically heterogeneous. Some interventions altered selected bacterial taxa, whereas others detected little or no change in overall microbial composition, and fecal or circulating SCFAs were not consistently quantified. Therefore, although resistance exercise may regulate gut microbial metabolism through muscle–gut communication, altered energy expenditure, inflammatory signaling, and dietary requirements, current evidence is insufficient to conclude that resistance training consistently increases SCFA production in humans.

Cross-sectional athlete studies provide additional, although indirect, evidence. Meng et al. [61] compared rugby players with male university students who were not majoring in physical education and reported differences in microbial composition and diversity together with higher fecal concentrations of SCFAs, including acetate, in the athletes. However, such findings should be interpreted cautiously because long-term training status, diet, body composition, and other lifestyle characteristics may contribute to the observed microbial phenotype. Thus, associations between resistance-oriented athletic training and SCFA concentrations do not by themselves establish a causal effect of resistance exercise. Overall, current evidence suggests a possible relationship between resistance exercise, the muscle–gut axis, and SCFA metabolism, but direct intervention studies with simultaneous assessment of microbial function and SCFA production remain necessary [62].

Exercise intensity represents another important source of variation in SCFA responses. More direct evidence for an intensity-dependent SCFA response has recently been provided by Reljic et al. [63]. In a pooled analysis of three randomized controlled trials involving 113 patients with obesity and metabolic syndrome, 12 weeks of low-volume high-intensity interval training combined with resistance exercise significantly increased total fecal SCFAs, acetate, propionate, and butyrate, whereas a moderate-intensity intervention of comparable structure did not produce significant changes. Notably, the mean blood lactate concentration achieved during training was strongly correlated with the increase in fecal SCFAs, suggesting that exercise intensity and the associated metabolic load may be important determinants of microbial SCFA responses. However, the relationship between exercise intensity and SCFA production does not appear to be simply linear. Zhang et al. [64] compared long-term moderate-intensity training with short-term high-intensity training while matching total exercise volume in mice. Long-term moderate-intensity training increased several SCFA-associated taxa, including Akkermansia, Prevotellaceae_NK3B31_group, Odoribacter, Alistipes, and Lactobacillus, and was accompanied by increased SCFA levels. In contrast, short-term high-intensity training increased potentially unfavorable taxa such as Staphylococcus and Bilophila without significantly increasing SCFAs.

In diet-induced obese mice, high-intensity exercise training altered the distal gut microbiota and significantly increased intestinal acetate, propionate, and butyrate concentrations. These changes were accompanied by improvements in energy metabolism, inflammation, and intestinal barrier function [65]. Similarly, studies in diabetic rats showed that both moderate-intensity continuous training (MICT) and HIIT increased SCFA levels after 8 weeks of intervention. The increases were accompanied by improved insulin resistance, gluconeogenic regulation, and lipid oxidation, while SCFA-related microbial pathways, including PWY-5676 and P163-PWY, were upregulated in the exercise groups [66]. These results indicate that different exercise intensities may converge on SCFA-related microbial metabolism despite producing distinct physiological demands.

However, the available evidence does not establish that greater exercise intensity necessarily produces greater SCFA increases. Comparisons of HIIT and MICT in metabolic dysfunction-associated steatotic liver disease (MASLD) have shown that both modalities can alter butyrate-associated bacterial populations and improve metabolic abnormalities, including insulin resistance and hepatic lipid accumulation [67]. Other studies have suggested that although HIIT can increase SCFAs, the magnitude of this response may be smaller than that induced by endurance training matched for energy expenditure [68]. One possible explanation is that prolonged endurance exercise provides a more sustained metabolic and intestinal stimulus, whereas HIIT produces a shorter but more pronounced metabolic perturbation.

Accordingly, SCFA responses should not be interpreted solely according to conventional exercise categories such as aerobic exercise, resistance training, or HIIT. Exercise intensity interacts with session duration, weekly frequency, total training volume, recovery, and intervention length. The biologically relevant variable may therefore be the integrated “exercise dose” rather than intensity alone. This concept may help explain why apparently similar exercise interventions have produced divergent SCFA responses across experimental and clinical studies.

### 3.3. Factors Modifying the Exercise–SCFA Relationship

Current evidence indicates that the effects of exercise on SCFA metabolism are strongly context dependent. Even within the same exercise modality, substantial differences can arise according to training duration, intensity, baseline fitness, metabolic health, diet, and the pre-existing gut microbial ecosystem. Individuals with T2DM, obesity, metabolic syndrome, or other disorders may begin with microbial communities and SCFA profiles that differ considerably from those of metabolically healthy individuals. Consequently, the magnitude and even the direction of microbial adaptation to exercise may vary among populations.

Exercise timing may represent another important modifier of the gut microbial response. Schönke et al. [69] compared treadmill training performed during the early versus late active phase in mice using otherwise comparable exercise protocols. Interestingly, only late-active-phase exercise increased the abundance of bacteria with SCFA-producing potential, whereas early-active-phase training did not induce the same microbial changes. These findings suggest that the effects of exercise on SCFA-related microbial metabolism may interact with host circadian rhythms and the diurnal activity of the gut microbiota. Martin et al. [70] examined healthy men spanning a broad range of aerobic exercise capacities while closely matching body composition and dietary habits. Aerobic exercise capacity was significantly associated with gut microbial composition and fecal SCFA profiles independently of relative nutrient intake. Notably, individuals with very high aerobic exercise capacity showed reduced microbial diversity, bacterial density, and functional richness, yet maintained relatively high fecal SCFA levels, with propionate and valerate showing particularly strong associations with exercise-capacity-related microbial features. These findings indicate that greater microbial diversity does not necessarily translate into greater SCFA-producing capacity and suggest that the functional organization of the microbial ecosystem may be more relevant than diversity alone.

Training status is another important consideration. Acute or short-term exercise exposure and long-term habitual training should not be regarded as equivalent biological stimuli. The reversibility of exercise-induced increases in butyrate-related bacteria and SCFA-associated functional genes following a return to sedentary behavior [53] suggests that continuous training may be required to maintain at least some of these microbial metabolic adaptations. Conversely, observations from competitive athletes [54,61] may reflect years of combined exercise, dietary, physiological, and lifestyle adaptation and therefore cannot be directly extrapolated to short-term exercise interventions.

The interpretation of SCFA measurements also requires caution. Fecal SCFA concentrations represent the balance between microbial production, microbial consumption, intestinal absorption, transit time, and fecal excretion [71]. Indeed, because the majority of SCFAs produced in the large intestine are rapidly absorbed, fecal concentrations largely represent the fraction remaining after intestinal uptake rather than the total amount produced [72]. Circulating concentrations are additionally influenced by splanchnic and hepatic metabolism as well as peripheral tissue utilization [73]. Therefore, neither fecal nor serum SCFA concentrations alone provide a direct measure of intestinal SCFA production. This methodological distinction may partly explain inconsistencies among exercise studies and is particularly important when determining whether physical activity truly enhances microbial SCFA synthesis.

Future studies should therefore move beyond simple comparisons of pre- and post-exercise SCFA concentrations. Integrating targeted SCFA profiling with metagenomic or metatranscriptomic analyses of SCFA-producing pathways, microbial functional genes, substrate utilization, and, where feasible, stable-isotope tracing would provide a more accurate assessment of SCFA production and flux [72,73]. Such approaches are essential for determining whether changes in SCFAs represent a reproducible metabolic adaptation to exercise and for identifying the exercise characteristics most likely to optimize SCFA-related benefits.

**Figure 3:**
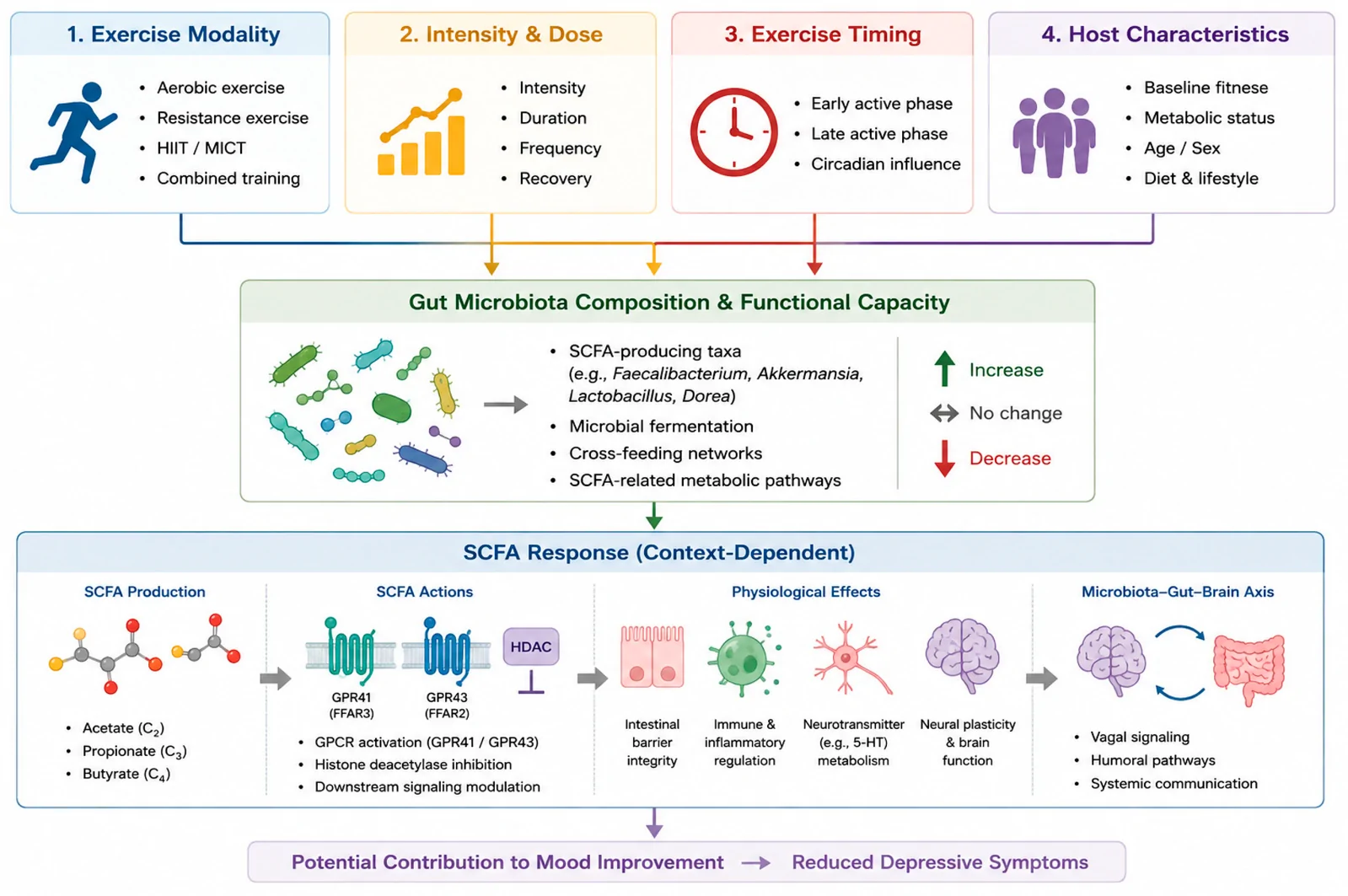
Context-dependent regulation of gut microbiota-derived SCFA production by physical activity.

Overall, current evidence suggests that physical activity is not a uniform enhancer of SCFA production but a context-dependent regulator of microbial fermentation. Appropriate and sustained exercise may favor microbial communities and metabolic networks that support acetate, propionate, and butyrate production, whereas the response can vary substantially according to exercise modality, dose, and host characteristics. Defining these boundary conditions is a necessary step before SCFAs can be considered a reliable mechanistic link between physical activity and downstream effects on the microbiota–gut–brain axis. The context-dependent regulation of gut microbiota-derived SCFA production by physical activity is summarized in Figure 3.

The available evidence regarding the effects of different exercise characteristics on SCFA-related microbial metabolism is summarized in Table 2.

## 4. Exercise-Mediated SCFA Regulation as a Potential Link to Depressive Symptoms

The evidence summarized above suggests that exercise can modify gut microbial composition and SCFA-related metabolism; however, whether these changes directly mediate the antidepressant effects of exercise remains incompletely established. Exercise-induced alterations in SCFA-producing or SCFA-associated taxa, including lactic acid bacteria, Faecalibacterium prausnitzii, Akkermansia, and Dorea, may influence microbial fermentation and the availability of acetate, propionate, and butyrate. These metabolites can subsequently interact with intestinal, immune, neural, and neuroendocrine pathways implicated in depression. Thus, SCFAs may represent an important metabolic interface linking exercise-induced remodeling of the gut microbiota to downstream changes along the microbiota–gut–brain axis. Nevertheless, given the heterogeneous SCFA responses to exercise described in Section 3, this pathway should be regarded as a context-dependent and potentially contributory mechanism rather than an established universal mediator of exercise-induced antidepressant effects.

### 4.1. Exercise-Induced Remodeling of SCFA-Associated Microbial Communities

Exercise can alter the abundance and metabolic activity of intestinal microorganisms associated with SCFA production. Previous studies have reported increases in SCFA-associated taxa, including lactic acid bacteria, Faecalibacterium prausnitzii, Akkermansia, and Dorea, following exercise interventions [74,75]. Such microbial remodeling may increase the fermentative potential of the intestinal ecosystem and favor SCFA-related metabolic activity. This possibility is particularly relevant to depression, in which alterations in SCFA-producing bacterial populations and microbial metabolic function have been reported.

However, changes in the relative abundance of SCFA-associated bacteria should not be equated directly with increased SCFA production. As discussed in Section 3, exercise-induced microbial responses vary according to exercise modality, intensity, dose, timing, training status, and host characteristics. Moreover, SCFA concentrations reflect not only microbial synthesis but also microbial cross-feeding, intestinal absorption, and host metabolism. Therefore, the potential antidepressant relevance of exercise-induced microbial remodeling is more appropriately interpreted as a shift in SCFA-related metabolic capacity rather than as evidence that exercise universally increases SCFA production.

### 4.2. Potential Downstream Pathways Linking Exercise-Regulated SCFAs to Depression

If exercise-induced microbial remodeling results in altered SCFA availability, several downstream pathways may connect these metabolic changes with depressive symptoms. Acetate, propionate, and butyrate can act as signaling metabolites through G protein-coupled receptors, particularly GPR41 (FFAR3) and GPR43 (FFAR2), while butyrate can also modulate gene expression through histone deacetylase inhibition. Through these mechanisms, SCFAs may influence intestinal barrier integrity, immune and inflammatory signaling, and neural function.

SCFAs may also contribute to the maintenance of intestinal epithelial integrity by regulating mucus production and barrier-associated signaling pathways, including AMP-activated protein kinase (AMPK) and myosin light chain-related mechanisms [76]. By strengthening the intestinal barrier and limiting the translocation of pro-inflammatory microbial products, SCFAs may reduce peripheral inflammatory signals capable of influencing the central nervous system. Butyrate can induce colonic tryptophan hydroxylase 1 (TPH1) expression and thereby increase peripheral serotonin (5-HT) synthesis [77]. However, peripheral 5-HT does not readily cross the blood–brain barrier and therefore cannot directly increase central 5-HT concentrations. Instead, gut-derived SCFAs and peripheral serotonergic signaling may influence central nervous system function indirectly through vagal afferent signaling, immune and endocrine pathways, or by modulating the availability of serotonin precursors such as tryptophan. This distinction is important when interpreting the potential contribution of SCFA-mediated peripheral 5-HT regulation to the antidepressant effects of exercise. Together with vagal and humoral communication, these mechanisms provide biological plausibility for an exercise–microbiota–SCFA–brain pathway.

Importantly, however, most available studies have examined individual components of this pathway rather than demonstrating the complete causal sequence from exercise to altered microbial SCFA production and subsequently to improvement in depressive symptoms. Evidence that exercise increases or modifies SCFAs should therefore not, by itself, be interpreted as proof that SCFAs mediate the antidepressant effects of exercise. Future studies combining exercise interventions with direct measurements of SCFA production and flux, microbiome functional analyses, SCFA receptor manipulation, metabolite supplementation or depletion, and behavioral outcomes will be necessary to establish the causal contribution of SCFAs to exercise-induced improvements in depression.

## 5. Conclusions and Future Perspectives

Accumulating evidence suggests that physical activity can modulate gut microbial SCFA metabolism, providing a potential metabolic link between exercise and the microbiota–gut–brain axis. However, exercise does not uniformly increase SCFA production. Instead, SCFA responses appear to depend on exercise modality, intensity, duration, frequency, training pattern, and timing, as well as host factors such as baseline fitness, metabolic status, diet, and gut microbial composition. Therefore, physical activity should be considered a context-dependent regulator of microbial SCFA metabolism rather than a simple enhancer of SCFA production.

Although SCFAs may contribute to the antidepressant effects of exercise through intestinal, immune, metabolic, and neural pathways, direct evidence establishing the complete causal sequence from exercise-induced SCFA changes to improvements in depressive symptoms remains limited. Future studies should therefore identify the exercise characteristics that optimize beneficial SCFA responses and distinguish changes in SCFA concentrations from actual production and metabolic flux. Integrating targeted metabolomics with multi-omics approaches, stable-isotope tracing, fecal microbiota transplantation, and SCFA-related pathway manipulation may help establish causality. Well-designed longitudinal studies and randomized controlled trials in individuals with depression are also needed to determine whether exercise-induced SCFA changes directly contribute to antidepressant outcomes.

## Figures and Tables

**Table 1:** Gut microbial alterations in depressive disorders and their potential relevance to SCFA metabolism.

Taxon	Alteration in Depression	Evidence from Systematic Reviews/Meta-Analyses	Major SCFA Relevance	Potential Implication for SCFA Metabolism
*Faecalibacterium*	↓	Consistently depleted in systematic reviews/meta-analyses [20,26]	Butyrate-producing [26,27]	Reduced abundance may indicate lower butyrate-producing potential
*Butyricicoccus*	↓	Consistently depleted in systematic reviews/meta-analyses [26]	Butyrate-producing [26]	Reduced abundance may indicate lower butyrate-producing potential
*Coprococcus*	↓	Consistently depleted in systematic reviews/meta-analyses [20,26]	Butyrate-producing/associated [27]	Reduced abundance may contribute to altered butyrate-related metabolism
Fusicatenibacter	↓	Consistently depleted in depressive disorder [26]	Acetate-associated fermentation [28]	May indicate reduced saccharolytic fermentation capacity
Romboutsia	↓	Consistently depleted in depressive disorder [26]	Acetate and other fermentation products [29]	May alter overall SCFA-related fermentation
Subdoligranulum	↓	Depleted in depressive disorder [26]	Butyrate [30]	May contribute to reduced butyrate-producing potential

Note: ↓, decreased abundance in depressive disorders. Evidence for depression-associated microbial alterations is primarily derived from systematic reviews and meta-analyses. The indicated SCFA-related characteristics reflect the known or proposed metabolic properties of the corresponding taxa and should not be interpreted as direct evidence of altered in vivo SCFA production or changes in the concentration or relative proportion of a specific SCFA.

**Table 2:** Effects of physical activity on gut microbiota-derived SCFA production.

Study	Population/Model	Exercise Modality	Exercise Dose	SCFA-Related Changes	Main Interpretation
Lambert et al. [48]	db/db mice	Low-intensity treadmill	6 weeks	↑ SCFA-associated taxa	Low-intensity aerobic exercise may enhance SCFA-producing potential
Allen et al. [53]	Humans	Endurance exercise	6 weeks	↑ Propionate; ↑ butyrate; changes reversed after detraining	SCFA-related adaptation may depend on sustained exercise
Reljic et al. [63]	Obesity/metabolic syndrome	HIIT + resistance exercise	12 weeks	↑ Total SCFAs; ↑ acetate; ↑ propionate; ↑ butyrate	SCFA response may be intensity dependent
Zhang et al. [64]	Mice	Long-term moderate-intensity vs. short-term high-intensity training	Exercise volume matched	Moderate-intensity training: ↑ SCFAs; high-intensity training: no significant increase	Higher intensity does not necessarily produce greater SCFA responses
Han et al. [66]	Diabetic rats	MICT vs. HIIT	8 weeks	Both MICT and HIIT: ↑ SCFAs	Different exercise intensities may converge on SCFA-related metabolism
Schönke et al. [69]	Mice	Treadmill exercise	Early vs. late active phase	Late-phase exercise: ↑ SCFA-producing potential	Exercise timing may modify SCFA-related microbial responses
Martin et al. [70]	Humans	Habitual aerobic exercise capacity	Cross-sectional	SCFA profile associated with aerobic exercise capacity	Baseline exercise capacity may modify the SCFA-related microbial phenotype

Abbreviations: HIIT, high-intensity interval training; MICT, moderate-intensity continuous training; SCFAs, short-chain fatty acids.

## Data Availability

The data supporting the findings of this study are available from the corresponding author upon.

## References

[1] World Health Organization (2026). Depressive Disorder (Depression).

[2] Wang D., Gui S., Pu J., Zhong X., Yan L., Li Z., Tao X., Yang D., Zhou H., Qiao R. (2025). PsycGM: A comprehensive database for associations between gut microbiota and psychiatric disorders. Mol. Psychiatry.

[3] National Institute for Health and Care Excellence (NICE) (2022). Depression in Adults: Treatment and Management.

[4] World Health Organization (2012). Physical Activity: Evidence-Based Recommendations for Management of Depression in Non-Specialized Health Settings.

[5] Stubbs B., Vancampfort D., Hallgren M., Firth J., Veronese N., Solmi M., Brand S., Cordes J., Malchow B., Gerber M. (2018). EPA guidance on physical activity as a treatment for severe mental illness: A meta-review of the evidence and Position Statement from the European Psychiatric Association (EPA), supported by the International Organization of Physical Therapists in Mental Health (IOPTMH). Eur. Psychiatry.

[6] Dalile B., Van Oudenhove L., Vervliet B., Verbeke K. (2019). The role of short-chain fatty acids in microbiota–gut–brain communication. Nat. Rev. Gastroenterol. Hepatol..

[7] Yang L.N., Li L., Wang S.N., Zhu L.J., Wei Y.P., Song H., He Y.T., Liu H. (2022). Research progress on the intestinal transport and absorption of short-chain fatty acids. J. Chin. Inst. Food Sci. Technol..

[8] Deleu S., Machiels K., Raes J., Verbeke K., Vermeire S. (2021). Short chain fatty acids and its producing organisms: An overlooked therapy for IBD?. eBioMedicine.

[9] Louis P., Flint H.J. (2009). Diversity, metabolism and microbial ecology of butyrate-producing bacteria from the human large intestine. FEMS Microbiol. Lett..

[10] Cook T.M., Gavini C.K., Jesse J., Aubert G., Gornick E., Bonomo R., Gautron L., Layden B.T., Mansuy-Aubert V. (2021). Vagal neuron expression of the microbiota-derived metabolite receptor, free fatty acid receptor (FFAR3), is necessary for normal feeding behavior. Mol. Metab..

[11] Skonieczna-Żydecka K., Grochans E., Maciejewska D., Szkup M., Schneider-Matyka D., Jurczak A., Łoniewski I., Kaczmarczyk M., Marlicz W., Czerwińska-Rogowska M. (2018). Faecal short chain fatty acids profile is changed in Polish depressive women. Nutrients.

[12] Bergersen L., Rafiki A., Ottersen O.P. (2002). Immunogold cytochemistry identifies specialized membrane domains for monocarboxylate transport in the central nervous system. Neurochem. Res..

[13] Resende W.R., Valvassori S.S., Reus G.Z., Varela R.B., Arent C.O., Ribeiro K.F., Bavaresco D.V., Andersen M.L., Zugno A.I., Quevedo J. (2013). Effects of sodium butyrate in animal models of mania and depression: Implications as a new mood stabilizer. Behav. Pharmacol..

[14] Lin H., Guo Q., Wen Z., Tan S., Chen J., Lin L., Chen P., He J., Wen J., Chen Y. (2021). The multiple effects of fecal microbiota transplantation on diarrhea-predominant irritable bowel syndrome (IBS-D) patients with anxiety and depression behaviors. Microb. Cell Factories.

[15] Koh A., De Vadder F., Kovatcheva-Datchary P., Bäckhed F. (2016). From dietary fiber to host physiology: Short-chain fatty acids as key bacterial metabolites. Cell Metab..

[16] Chen H., Xu Y., Qiu J., Guan Z., Yu S., Ding Y. (2026). Differential regulatory mechanisms of gut-derived SCFAs (acetate, propionate, butyrate) on the pulmonary TLR2 signaling pathway and their roles in the inflammatory balance of bacterial pneumonia. Eur. J. Med. Res..

[17] Hurst N.R., Kendig D.M., Murthy K.S., Grider J.R. (2014). The short chain fatty acids, butyrate and propionate, have differential effects on the motility of the guinea pig colon. Neurogastroenterol. Motil..

[18] Facchin S., Calgaro M., Savarino E.V. (2025). Rethinking short-chain fatty acids: A closer look at propionate in inflammation, metabolism, and mucosal homeostasis. Cells.

[19] Nshanian M., Gruber J.J., Geller B.S., Chleilat F., Lancaster S.M., White S.M., Alexandrova L., Camarillo J.M., Kelleher N.L., Zhao Y. (2025). Short-chain fatty acid metabolites propionate and butyrate are unique epigenetic regulatory elements linking diet, metabolism and gene expression. Nat. Metab..

[20] McGuinness A.J., Davis J.A., Dawson S.L., Loughman A., Collier F., O’Hely M., Berk M., Staudacher H.M., Jacka F.N. (2022). A systematic review of gut microbiota composition in observational studies of major depressive disorder, bipolar disorder and schizophrenia. Mol. Psychiatry.

[21] Reigstad C.S., Salmonson C.E., Rainey J.F., Szurszewski J.H., Linden D.R., Sonnenburg J.L., Farrugia G., Kashyap P.C. (2014). Gut microbes promote colonic serotonin production through an effect of short-chain fatty acids on enterochromaffin cells. FASEB J..

[22] Rajkumar H., Kumar M., Das N., Kumar S.N., Challa H.R., Nagpal R. (2015). Effect of probiotic Lactobacillus salivarius UBL S22 and prebiotic fructo-oligosaccharide on serum lipids, inflammatory markers, insulin sensitivity, and gut bacteria in healthy young volunteers: A randomized controlled single-blind pilot study. J. Cardiovasc. Pharmacol. Ther..

[23] García-Montero C., Fraile-Martinez O., Gomez-Lahoz A.M., Pekarek L., Castellanos A.J., Noguerales-Fraguas F., Coca S., Guijarro L.G., García-Honduvilla N., Asúnsolo A. (2021). Nutritional components in Western diet versus Mediterranean diet at the gut microbiota-immune system interplay. Implications for health and disease. Nutrients.

[24] Behrens L.M.P., Gasparotto J., Rampelotto P.H., Escalona M.A.R., Silva L.d.S.d., Carazza-Kessler F.G., Barbosa C.P., Campos M.S., Dorn M., Gelain D.P. (2024). Sodium propionate oral supplementation ameliorates depressive-like behavior through gut microbiome and histone 3 epigenetic regulation. J. Nutr. Biochem..

[25] Van de Wouw M., Boehme M., Lyte J.M., Wiley N., Strain C., O’Sullivan O., Clarke G., Stanton C., Dinan T.G., Cryan J.F. (2018). Short-chain fatty acids: Microbial metabolites that alleviate stress-induced brain-gut axis alterations. J. Physiol..

[26] Gao M., Wang J., Liu P., Tu H., Zhang R., Zhang Y., Sun N., Zhang K. (2023). Gut microbiota composition in depressive disorder: A systematic review, meta-analysis, and meta-regression. Transl. Psychiatry.

[27] Duncan S.H., Barcenilla A., Stewart C.S., Pryde S.E., Flint H.J. (2002). Acetate utilization and butyryl coenzyme A (CoA):acetate-CoA transferase in butyrate-producing bacteria from the human large intestine. Appl. Environ. Microbiol..

[28] Takada T., Kurakawa T., Tsuji H., Nomoto K. (2013). *Fusicatenibacter saccharivorans* gen. nov., sp. nov., isolated from human faeces. Int. J. Syst. Evol. Microbiol..

[29] Gerritsen J., Hornung B., Renckens B., van Hijum S.A.F.T., Martins dos Santos V.A.P., Rijkers G.T., Schaap P.J., de Vos W.M., Smidt H. (2017). Genomic and functional analysis of Romboutsia ilealis CRIBT reveals adaptation to the small intestine. PeerJ.

[30] Soto-Martin E.C., Warnke I., Farquharson F.M., Christodoulou M., Horgan G., Derrien M., Faurie J.-M., Flint H.J., Duncan S.H., Louis P. (2020). Vitamin biosynthesis by human gut butyrate-producing bacteria and cross-feeding in synthetic microbial communities. mBio.

[31] Xie Y.J. (2023). The Role and Mechanisms of the Gut Microbiota in the Development and Treatment of Depression. Ph.D. Thesis.

[32] Detka J., Głombik K. (2021). Insights into a possible role of glucagon-like peptide-1 receptor agonists in the treatment of depression. Pharmacol. Rep..

[33] Lal S., Kirkup A.J., Brunsden A.M., Thompson D.G., Grundy D. (2001). Vagal afferent responses to fatty acids of different chain length in the rat. Am. J. Physiol.-Gastrointest. Liver Physiol..

[34] Buckley M.M., O’Brien R., Brosnan E., Ross R.P., Stanton C., Buckley J.M., O’mAlley D. (2020). Glucagon-like peptide-1 secreting L-cells coupled to sensory nerves translate microbial signals to the host rat nervous system. Front. Cell. Neurosci..

[35] Chen L., Tao F.B., Huang K. (2024). Role of short-chain fatty acids in neuropsychiatric disorders mediated by the microbiota–gut–brain axis. Acta Nutr. Sin..

[36] Liu Z.Y., Zou M.S., Han Y.S., Zhao H., He H., Tang L., Wang Y. (2025). Interaction between the gut microbiota and the P2X7R/NLRP3 signaling pathway: Neuroinflammatory and metabolic regulatory mechanisms in depression and related traditional Chinese medicine interventions. Chin. Tradit. Herb. Drugs.

[37] Goldsmith D.R., Rapaport M.H., Miller B.J. (2016). A meta-analysis of blood cytokine network alterations in psychiatric patients: Comparisons between schizophrenia, bipolar disorder and depression. Mol. Psychiatry.

[38] Li J.M., Yu R., Zhang L.P., Wen S.-Y., Wang S.-J., Zhang X.-Y., Xu Q., Kong L.-D. (2019). Dietary fructose-induced gut dysbiosis promotes mouse hippocampal neuroinflammation: A benefit of short-chain fatty acids. Microbiome.

[39] Li X. (2021). The Role and Underlying Mechanisms of Short-Chain Fatty Acids in Glucose and Lipid Metabolism in First-Episode Schizophrenia. Ph.D. Thesis.

[40] Lu S.C., Zheng N., Wang J.Q., Zhang Y.D. (2024). Research progress on the biological functions of short-chain fatty acids. Chin. J. Anim. Nutr..

[41] Arpaia N., Campbell C., Fan X., Dikiy S., Van Der Veeken J., DeRoos P., Liu H., Cross J.R., Pfeffer K., Coffer P.J. (2013). Metabolites produced by commensal bacteria promote peripheral regulatory T-cell generation. Nature.

[42] Bai C., Guo R.J., Wang J.Y., Wang Y.F., Ao Y.T. (2024). Exploring the pathogenesis of liver depression and spleen deficiency in depression based on short-chain fatty acids and the gut microbiota. World Chin. Med..

[43] Wu J.J. (2020). Protective Effects of Xiaoyao San on Neuroinflammation and the Colonic Epithelial Barrier in Mice with Gut Microbiota Dysbiosis. Ph.D. Thesis.

[44] Yao J., Yang Y.T., Ai Q.D., Lin M.Y., Sun Y., Long J.P., Yang S.W., Chen N.H. (2023). Research progress on neurotransmitter function and the pathogenesis of depression. Chin. Pharmacol. Bull..

[45] Sun J., Wang F., Hu X., Yang C., Xu H., Yao Y., Liu J. (2018). Clostridium butyricum attenuates chronic unpredictable mild stress-induced depressive-like behavior in mice via the gut–brain axis. J. Agric. Food Chem..

[46] Sun J., Wang F., Hong G., Pang M., Xu H., Li H., Tian F., Fang R., Yao Y., Liu J. (2016). Antidepressant-like effects of sodium butyrate and its possible mechanisms of action in mice exposed to chronic unpredictable mild stress. Neurosci. Lett..

[47] Wang S., Chen J., Gao C., Zhou Y., Wei Z., Liang Z. (2026). Effects of aerobic exercise on cognition, sleep, and mood in healthy adults: A systematic review and meta-analysis of randomized controlled trials. Front. Hum. Neurosci..

[48] Lambert J.E., Myslicki J.P., Bomhof M.R., Belke D.D., Shearer J., Reimer R.A. (2015). Exercise training modifies gut microbiota in normal and diabetic mice. Appl. Physiol. Nutr. Metab..

[49] Torquati L., Gajanand T., Cox E.R., Willis C.R.G., Zaugg J., Keating S.E., Coombes J.S. (2023). Effects of exercise intensity on gut microbiome composition and function in people with type 2 diabetes. Eur. J. Sport Sci..

[50] Sohail M.U., Yassine H.M., Sohail A., Al Thani A. (2019). Impact of physical exercise on gut microbiome, inflammation, and the pathobiology of metabolic disorders. Rev. Diabet. Stud..

[51] Abraham D., Feher J., Scuderi G.L., Szabo D., Dobolyi A., Cservenak M., Juhasz J., Ligeti B., Pongor S., Gomez-Cabrera M.C. (2019). Exercise and probiotics attenuate the development of Alzheimer’s disease in transgenic mice: Role of microbiome. Exp. Gerontol..

[52] Li Y., Zafar S., Ibrahim R.M.S., Chi H.-L., Xiao T., Xia W.-J., Li H.-B., Kang Y.-M. (2021). Exercise and food supplement of vitamin C ameliorate hypertension through improvement of gut microflora in the spontaneously hypertensive rats. Life Sci..

[53] Allen J.M., Mailing L.J., Niemiro G.M., Moore R., Cook M.D., White B.A., Holscher H.D., Woods J.A. (2018). Exercise alters gut microbiota composition and function in lean and obese humans. Med. Sci. Sports Exerc..

[54] Scheiman J., Luber J.M., Chavkin T.A., MacDonald T., Tung A., Pham L.-D., Wibowo M.C., Wurth R.C., Punthambaker S., Tierney B.T. (2019). Meta-omics analysis of elite athletes identifies a performance-enhancing microbe that functions via lactate metabolism. Nat. Med..

[55] Lv X., Yang M., Wang F., Wang Y., He X., Yi J., Wang L., Liao S. (2020). The effect of Baduanjin on intestinal flora in patients with prediabetes mellitus: Study protocol for a randomized controlled trial. Medicine.

[56] Wei W., Zhang Q., Huang Y.F., Zhai L., Liu Y.H., Ma C., Huang S. (2022). Effects of different exercise modalities on the gut microbiota and short-chain fatty acids in mice with type 2 diabetes. J. Guangxi Med. Univ..

[57] Zhao Y.H., Wang W.X., Wang M.D., Gao F., Hu C., Cui B.W., Yu W.L., Ren H. (2025). Effects of an individualized progressive exercise prescription on anxiety among university students. Chin. J. Sports Med..

[58] Bycura D., Santos A.C., Shiffer A., Kyman S., Winfree K., Sutliffe J., Pearson T., Sonderegger D., Cope E., Caporaso J.G. (2021). Impact of different exercise modalities on the human gut microbiome. Sports.

[59] Ma C., Huang Y.F., Zhai L., Liu Y.H., Zhang Q., Huang S. (2020). Effects of different exercise modalities on serum short-chain fatty acids in mice with type 2 diabetes. West China Med. J..

[60] Wagner A., Kapounková K., Struhár I. (2024). The relationship between the gut microbiome and resistance training: A rapid review. BMC Sports Sci. Med. Rehabil..

[61] Meng X.F., Li S., Feng G.S., Meng Y., Zhang H., Sha J. (2024). Characteristics of the gut microbiota and short-chain fatty acid levels in male rugby players and their anti-inflammatory effects. Chin. J. Microecol..

[62] Liu Y.Y., Qiu J.Q. (2023). Research progress on exercise-induced regulation of short-chain fatty acids in delaying aging and its underlying mechanisms. Chin. Bull. Life Sci..

[63] Reljic D., Frenkel M.O., Herrmann H.J., Neurath M.F., Zopf Y. (2025). Exercise improves gut microbial metabolites in an intensity-dependent manner: A pooled analysis of randomized controlled trials. Gut Microbes.

[64] Zhang Y., Wang C., Lang H., Yu H., Zhou M., Rao X., Zhang Q., Yi L., Zhu J., Mi M. (2024). The contrasting effects of two distinct exercise training modalities on exhaustive exercise-induced muscle damage in mice may be associated with alterations in the gut microbiota. Int. J. Mol. Sci..

[65] Denou E., Marcinko K., Surette M.G., Steinberg G.R., Schertzer J.D. (2016). High-intensity exercise training increases the diversity and metabolic capacity of the mouse distal gut microbiota during diet-induced obesity. Am. J. Physiol.-Endocrinol. Metab..

[66] Han Y., Quan H., Ji W., Tian Q., Liu X., Liu W. (2024). Moderate-intensity continuous training and high-intensity interval training alleviate glycolipid metabolism through modulation of gut microbiota and their metabolite SCFAs in diabetic rats. Biochem. Biophys. Res. Commun..

[67] Deng D., Xu L., Liu Y., Li C., Jiang Q., Shi J., Feng S., Lin Y. (2025). HIIT versus MICT in MASLD: Mechanisms mediated by gut-liver axis crosstalk, mitochondrial dynamics remodeling, and adipokine signaling attenuation. Lipids Health Dis..

[68] Wang Y., Bai S., Yang T., Guo J., Zhu X., Dong Y. (2025). Impact of exercise-induced alterations on gut microbiota diversity and composition: Comparing effects of different training modalities. Cell Regen..

[69] Schönke M., Ying Z., Kovynev A., In het Panhuis W., Binnendijk A., van der Poel S., Pronk A.C.M., Streefland T.C.M., Hoekstra M., Kooijman S. (2023). Time to run: Late rather than early exercise training in mice remodels the gut microbiome and reduces atherosclerosis development. FASEB J..

[70] Martin A.M., Bonneau M., Orfila L., Horeau M., Hazon M., Demay R., Lecommandeur E., Boumpoutou R., Guillotel A., Guillemot P. (2025). Atypical gut microbial ecosystem from athletes with very high exercise capacity improves insulin sensitivity in mice. Cell Rep..

[71] Mukhopadhya I., Louis P. (2025). Gut microbiota-derived short-chain fatty acids and their role in human health and disease. Nat. Rev. Microbiol..

[72] Sakata T. (2019). Pitfalls in short-chain fatty acid research: A methodological review. Anim. Sci. J..

[73] Pouteau E., Nguyen P., Ballèvre O., Krempf M. (2003). Production rates and metabolism of short-chain fatty acids in the colon and whole body using stable isotopes. Proc. Nutr. Soc..

[74] Liu Y.Y., Qiu J.Q. (2023). Exercise and gut microbiota-derived metabolites: The metabolic regulatory role of short-chain fatty acids in type 2 diabetes. Chin. J. Sports Med..

[75] Xu L., Ni Z., Zhang Y. (2022). Exercise, gut microbiota-derived metabolites, and short-chain fatty acids in the regulation of skeletal muscle metabolism. Chin. J. Biochem. Mol. Biol..

[76] Kong J.D., Xu X.R., Chen S.J., Zhu L. (2024). Effects of gut microbiota-derived short-chain fatty acids on the improvement of exercise performance. Prog. Physiol. Sci..

[77] Shu X.L. (2022). Effects and Underlying Mechanisms of Xylo-Oligosaccharides in Alleviating Depressive-like Behaviors in Mice. Ph.D. Thesis.

